# Influence of Human Milk on Very Preterms’ Gut Microbiota and Alkaline Phosphatase Activity

**DOI:** 10.3390/nu13051564

**Published:** 2021-05-06

**Authors:** Juliana Morais, Cláudia Marques, Ana Faria, Diana Teixeira, Inês Barreiros-Mota, Catarina Durão, João Araújo, Shámila Ismael, Sara Brito, Manuela Cardoso, Israel Macedo, Esmeralda Pereira, Teresa Tomé, Conceição Calhau

**Affiliations:** 1 Faculdade de Ciências Médicas|NOVA Medical School, Universidade NOVA de Lisboa, 1169-056 Lisboa, Portugal; juliana.morais@nms.unl.pt (J.M.); claudia.sofia.marques@nms.unl.pt (C.M.); ana.faria@nms.unl.pt (A.F.); diana.teixeira@nms.unl.pt (D.T.); ines.mota@nms.unl.pt (I.B.-M.); catarina.durao@nms.unl.pt (C.D.); joaoricardo.araujo@nms.unl.pt (J.A.); shamila.ismael@nms.unl.pt (S.I.); 2 CHRC-Comprehensive Health Research Centre, CEDOC-Chronic Diseases Research Center, Faculdade de Ciências Médicas|NOVA Medical School, Universidade NOVA de Lisboa, 1169-056 Lisboa, Portugal; 3 CINTESIS-Center for Health Technology Services Research, Faculdade de Ciências Médicas|NOVA Medical School, Universidade NOVA de Lisboa, 1169-056 Lisboa, Portugal; 4 NOVA Medical School, Unidade Universitária Lifestyle Medicine José de Mello Saúde, 1169-056 Lisboa, Portugal; 5 EPIUnit-Institute of Public Health, Universidade do Porto, 4050-600 Porto, Portugal; 6 Pediatrics Department, Maternidade Dr. Alfredo da Costa, Centro Hospitalar Universitário de Lisboa Central, 2890-495 Lisboa, Portugal; sarabri@gmail.com (S.B.); israeljmacedo@gmail.com (I.M.); esmeralda.pereira@chlc.min-saude.pt (E.P.); teresatome@netcabo.pt (T.T.); 7 Nutrition and Dietetics Unit, Maternidade Dr. Alfredo da Costa, Centro Hospitalar Universitário de Lisboa Central, 2890-495 Lisboa, Portugal; mariamanuelacardoso.mc@gmail.com

**Keywords:** alkaline phosphatase, donor human milk, formula, gut microbiota, mother’s own milk, very preterm neonates

## Abstract

The FEEDMI Study (NCT03663556) evaluated the influence of infant feeding (mother’s own milk (MOM), donor human milk (DHM) and formula) on the fecal microbiota composition and alkaline phosphatase (ALP) activity in extremely and very preterm infants (≤32 gestational weeks). In this observational study, preterm infants were recruited within the first 24 h after birth. Meconium and fecal samples were collected at four time points (between the 2nd and the 26th postnatal days. Fecal microbiota was analyzed by RT-PCR and by 16S rRNA sequencing. Fecal ALP activity, a proposed specific biomarker of necrotizing enterocolitis (NEC), was evaluated by spectrophotometry at the 26th postnatal day. A total of 389 fecal samples were analyzed from 117 very preterm neonates. Human milk was positively associated with beneficial bacteria, such as *Bifidobacterium*, *Bacteroides ovatus*, and *Akkermancia muciniphila*, as well as bacterial richness. Neonates fed with human milk during the first week of life had increased *Bifidobacterium* content and fecal ALP activity on the 26th postnatal day. These findings point out the importance of MOM and DHM in the establishment of fecal microbiota on neonates prematurely delivered. Moreover, these results suggest an ALP pathway by which human milk may protect against NEC.

## 1. Introduction

Very preterm infants (<32 weeks of gestational age) present an intestinal microbiota significantly different from that of full-term infants [1], being characterized by a highly permeable intestine surface and a marked vulnerability to dysbiosis [2]. Increasing data suggests that the type of infant feeding significantly influences the preterm infants’ development as well as their microbiota [3]. Mother’s own milk (MOM) is the gold standard for preterm infants’ nutrition during hospitalization [4,5], leading them to develop a microbiota resembling that of term infants [6]. When MOM is not available, or it is insufficient, pasteurized donor human milk (DHM) is recommended [7]. Bovine-based preterm formulas are the final alternative for preterm infants’ nutrition [7]. Even though formula is associated with increased in-hospital weight gain and better growth, it is also linked with a higher risk of necrotizing enterocolitis (NEC) [8,9], a very common cause of morbidity and mortality in preterm infants, closely associated with gut microbiota [10].

Recently, the intestinal isoform of alkaline phosphatase (ALP)—an enzyme that reduces lipopolysaccharide-mediated inflammation [11]—was suggested to be a specific biomarker for NEC [12]. Since preterm neonates have reduced ALP when compared to early-term neonates, it has been suggested that the lack of ALP activity could increase the risk of inflammation and progression to NEC [13]. The modulation of ALP activity has been poorly studied. In fact, the association between the triad of feeding type, ALP activity, and gut microbiota has never been evaluated in hospitalized preterm neonates.

Thus, the aim of this study was to evaluate the impact of different types of infant feeding (MOM, DHM, and formula) during the first 4 weeks of life on the fecal microbiota and on ALP activity of very and extremely preterm neonates.

## 2. Materials and Methods

### 2.1. Study Design and Participants

The FEEDMI study is an observational longitudinal study conducted at the Neonatal Intensive Care Unit (NICU) of Maternidade Dr. Alfredo da Costa and Faculdade de Ciências Médicas|NOVA Medical School, Universidade NOVA de Lisboa between May 2017 and April 2019. This study, approved by both the hospital and faculty ethics committees, is registered on the ClinicalTrials.gov platform (NCT03663556) and was conducted in accordance to the ethical principles expressed in the Declaration of Helsinki, the Portuguese law, and Good Clinical Practice guidelines.

Extremely and very preterm neonates included in this study were recruited according to their admission in the NICU within the first 24 h post-birth. Eligible neonates were born at a gestational age of < 32 weeks and had no congenital malformations or metabolic diseases. Signed written informed consent was obtained for each newborn after explaining the study protocol to their legal representatives. The detailed study protocol was previously published [14].

### 2.2. Enteral Nutrition Protocol

Exclusive human milk (MOM or DHM) was the recommended procedure. When MOM was insufficient or not available, DHM could be used for neonates until 35 weeks’ postmenstrual age. Mothers were advised to collect milk with breast pumps every 3 h either in the hospital or at home. The MOM samples received were identified by date and time of collection. MOM was frozen and stored, but never pasteurized. DHM was frozen and submitted to Holder pasteurization (62.5 °C for 30 min) in the maternity human milk bank. For each neonate, MOM samples were analyzed once a week and DHM was analyzed after the pasteurization using a mid-infrared human milk analyzer (Miris AB, Uppsala, Sweden). Formula was used if MOM was not sufficient and if the DHM stock was limited. Nutritional daily plans were prescribed by physicians in collaboration with a nutritionist.

### 2.3. Clinical Data Collection

Detailed sociodemographic, perinatal, and postnatal clinical data were collected through medical records. Clinical intrapartum and postpartum data included newborn’s somatometric progression, antibiotic exposure and its duration, number of total days of hospitalization, and other outcomes related to the preterm clinical evolution. Weight, length, and cephalic perimeter Z-scores at birth were determined using the growth curves obtained by Fenton [15]. Additionally, type of infant feeding was recorded daily to select the most representative (>50%) type of feeding received during the study period.

### 2.4. Fecal Sample Collection and DNA Extraction

Meconium was collected within the first 72 h after birth and subsequent fecal samples were collected weekly over the first four weeks of life. All samples were collected by the nursing team of the NICU. Bacterial DNA was extracted and purified from all fecal samples using a NZY Tissue gDNA Isolation Kit (NZYtech, Lisbon, Portugal) as previously described [16].

### 2.5. Quantitative Analysis of Fecal Microbiota by RT-PCR

Specific bacterial populations were analyzed in duplicate by quantitative real-time PCR (RT-PCR) using LightCycler^®^ (Roche Applied Science, Indianapolis, ID, USA). Specific microorganisms were assessed based on previous studies regarding preterm gut microbiota composition and health [17,18,19,20]. Two phyla (*Bacteroidetes* and *Firmicutes*), one class (*γ-Proteobacteria*), four genera (*Lactobacillus*, *Bifidobacterium*, *Bacteroides,* and *Enterococcus*), and one species (*Escherichia coli*) were analyzed in preterm neonates’ samples. Primers sequences used to target bacterial 16S rRNA genes are described in Table 1. Microbiota results are expressed as log10 16S rRNA gene copies/10 ng of DNA. All of the analyses were conducted using the appropriate negative controls, as previously described [21].

### 2.6. Microbial 16S rRNA Sequence Analysis

Due to the limited volume of the fecal samples of preterm infants, a sub-set of fecal samples collected in the fourth week of life was analyzed by 16S rRNA sequencing. The fecal samples of preterm infants fed with MOM (*n* = 40), DHM (*n* = 14), and formula (*n* = 10) were investigated. Libraries were processed and sequenced following the 16S Metagenomic Sequencing Library Preparation protocol from illumina (illumina; San Diego, CA, USA). Primers used to capture the region V3–V4 of the bacterial 16S region (primers 515F: GTGYCAGCMGCCGCGGTAA; 806R: GGACTACNVGGGTWTCTAAT) were previously described by Walters and colleges (2015) [24]. The samples were pooled and loaded into the illumina MiSeq System and sequenced using a 280-multiplex approach on a 2 × 250 bp run, according to manufacturer’s procedures [24]. For all sequencing reads, QIIME 2.11 was used with default parameters for demultiplexing, quality filtering, and clustering reads into OTUs. The Greengenes database was used for taxonomy assignment. Gut microbial diversity was evaluated by Shannon index, and gut microbial richness was measured by Chao1.

### 2.7. Alkaline Phosphatase Activity Assay

ALP activity was assessed through dephosphorylation of p-nitrophenyl phosphate (pNPP) in p-nitrophenol, which results in a color change that was measured by absorbance spectrophotometry. The protocol was performed as previously described by Calhau (2000) [25] and Malo (2015) [26] with some modifications. Briefly, 50 µL of “extraction buffer” (10 mM Tris-HCl and 1 mM MgCl2, pH 8.0) was added to 1 mg of fecal samples for homogenization and the sample was centrifuged at 10,000× *g* for 20 min. Using a 1:50 ratio, the “dilution buffer” (200 mM Tris-base and 1 mM MgCl2, pH 10.4) was added to the supernatant containing ALP, along with 10 µL of pNPP (5 mM), and the sample was incubated for 2 min at 37 °C. The reaction was stopped with 5 mL of cold NaOH (0.02 M). Absorbance was read at 410 nm and results were expressed as U ALP/g stool.

### 2.8. Statistical Analysis

Statistical analysis was performed by SPSS^®^ software, v25 (IBM SPSS Statistics corporation, Chicago, IL, USA). The normality of the data was checked using the Kolmogorov–Smirnov test. Comparisons between groups were performed using the t-test, Mann–Whitney test, or Fisher’s exact test. Multivariate linear regression models adjusted for gestational age, mode of delivery, Z-scores’ growth parameters (weight, length, and cephalic perimeter at birth), and infant’s antibiotherapy were fitted to study the association between infant feeding (independent variable) and microbiota composition of very preterm neonates at the 26th postnatal day (dependent variables). The sequencing data of fecal samples was analyzed using MicrobiomeAnalyst [27]. Alpha-diversity of the fecal microbiota was assessed through Chao1 and the Shannon index for microbial richness and diversity index, respectively. Beta-diversity was performed using PERMANOVA. The differences in relative abundance of fecal bacterial taxa between the three feeding types was assessed by Kruskal–Wallis. Differences were considered statistically significant when *p* < 0.05. Data are presented as mean ± SD.

## 3. Results

### 3.1. Clinical Characterization of the Preterm Neonates

From the 159 preterm neonates recruited consecutively at the NICU, 117 were eligible for the study with forty-two were excluded: 14 for not meeting the inclusion criteria, 13 due to early death, 7 for having nonconformities in their fecal samples, 4 for early discharge, 3 were transferred to another hospital, and 1 for unknown perinatal factors. The 117 eligible neonates were born with a gestational age between 25 and 31 weeks (28.6 ± 1.9) with birthweight ranging from 455 to 2020 g (1177 ± 419). Most of the neonates were male (56%) and delivered by cesarean-section (62%). Enteral feeding was introduced between the 1st and 6th postnatal day and exclusive enteral feeding was achieved at 15 ± 8 postnatal days. Preterm neonates were either fed with their MOM, DHM, and/or infant formula. During the 26 days of the study, MOM was the main feeding type for most neonates (*n* = 75, 64.1%), representing more than 50% of the total feedings. Twenty neonates (17.1%) were fed predominantly with DHM and 13 (11.1%) with infant formula. In some cases, neonates received mixed feeding of MOM + DHM (*n* = 7, 6.0%; 29.2 ± 1.2 weeks of gestational age; 1389 ± 153 g birth weight) or MOM + formula (*n* = 1, 0.85%; 31 weeks of gestational age; 1800 g birth weight). Due to specific clinical conditions, one preterm neonate was fed mainly by parenteral feeding, reaching exclusive enteral feeding at the 48th postnatal day and, therefore, was excluded from our analysis.

Extremely preterm neonates, those with lower birth weight, length, or cephalic perimeter, were preferably fed with MOM and DHM rather than formula (Table 2). Neonates fed predominantly with formula showed a greater weight gain, fewer days of antibiotherapy, and lower total days of hospitalization compared to MOM and DHM (Table 2).

### 3.2. Impact of Feeding on Intestinal Bacterial Establishment in Premature Neonates

**Figure 1:**
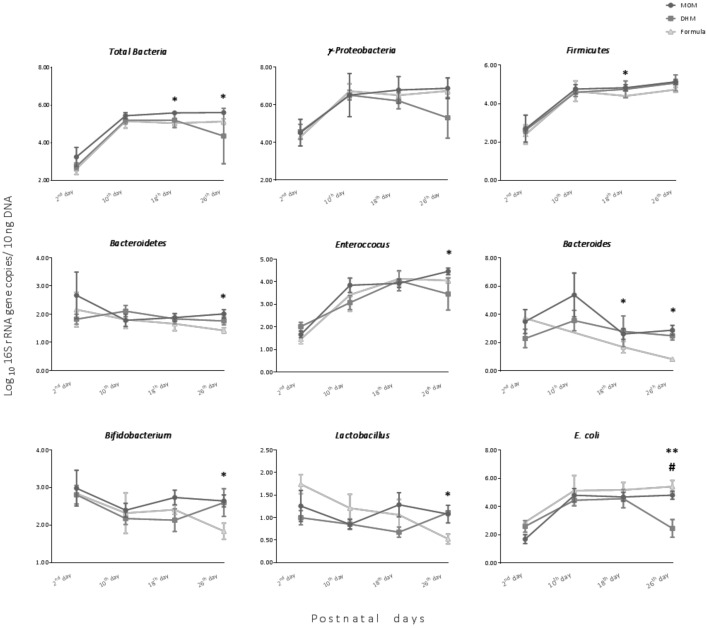
Microbiota of neonates during the study period according to the predominant feeding received in the 8 days preceding each fecal collection. The means and standard mean errors of relative abundance are shown. * MOM vs. formula; ** MOM vs. DHM; ^#^ DHM vs. formula. Comparisons between feeding groups in each time point were performed using t-test or Mann–Whitney test, taking in account the distribution of the variables. Differences were statistically significant when *p* < 0.05. MOM, mother’s own milk. DHM, donor human milk.

A total of 389 fecal samples were collected weekly during the first four weeks of life with an average postnatal days of 2 ± 0.9 (corresponding to meconium samples), 10 ± 1.9, 18 ± 2.1, and 26 ± 2.5 days. Feeding trends over time were assessed week-by-week considering the predominant feeding type (Figure 1). In general, the level of total bacteria increased significantly during the first week of life. At day 26 of life, infants fed with MOM had higher levels of total bacteria *Bacteroidetes*, *Enterococcus*, *Bacteroides*, *Bifidobacterium,* and *Lactobacillus* when compared to that of formula fed infants (Figure 1).

Table 3 shows the cumulative effect of the predominant feeding type received during the first 26 days on preterm microbiota composition (total bacteria, *Firmicutes,* and *Bifidobacterium*; no other differences were observed). Compared to MOM, DHM and formula feeding promoted lower levels of total bacteria in very preterm neonates at the 26th postnatal day when adjusted for gestational age, mode of delivery, and for multivariable-adjusted model (Table 3). Generally, MOM promoted more *Bifidobacterium* content, and this difference was significant after adjusting for gestational age when comparing to that of DMH and after adjusting for gestational age and for multivariable model when comparing to that of formula (Table 3). After adjustment for multivariable model, *Firmicutes* were fewer in DHM and formula fed neonates (Table 3).

### 3.3. Gut Microbiota Profile at the 26th Postnatal Day According to Feeding Types

**Figure 2:**
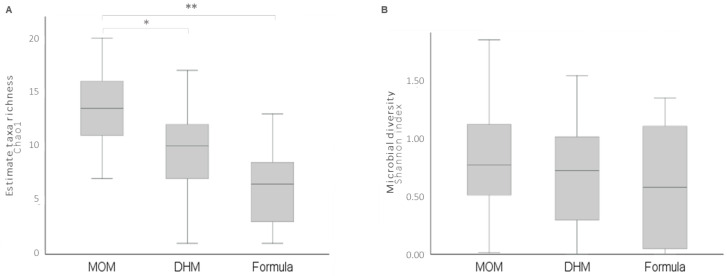
(**A**) Microbial richness (Chao1 index) and (**B**) diversity (Shannon index) of fecal microbiota in preterm infants according to feeding types. * *p* < 0.05 and ** *p* < 0.01. MOM, mother’s own milk. DHM, donor human milk.

Considering the results from 16S rRNA sequencing, three samples were excluded from analysis due their low sequencing reads, leaving 61 samples (MOM = 39, DHM = 14, Formula = 8). Preterm neonates fed with MOM had an increased microbial richness (Chao1 index) compared to that of DHM (*p* = 0.011) and formula (*p* < 0.001) (Figure 2). No differences were found between groups regarding Shannon index

**Figure 3:**
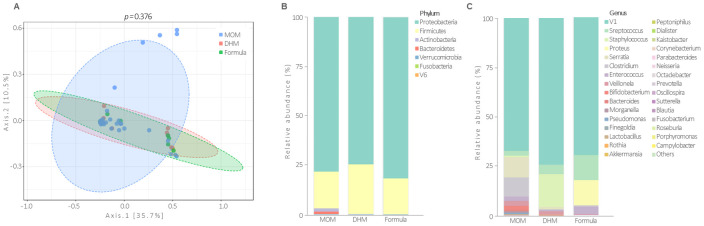
Microbiota composition in the fecal samples of preterm infants according to the predominant feeding type received during the first 26 days of life (mother’s own milk, MOM; donor human milk, DHM; formula). Principal coordinates analyses (PCoA, Bray-Curtis index) (**A**), each point represents one individual, and each circle represents a microbial community. Relative abundances of the dominant phylum (**B**) and genus (**C**).

When assessing beta-diversity, no differences were found on the fecal microbiota profiles between infants fed with MOM, DHM, and formula (Figure 3A). Differences in the relative abundance of phylum *Proteobacteria*, *Bacteroidetes*, *Verrucomicrobia* (*p* < 0.05 and *q* < 0.01) among the three types of feeding (Figure 3B) were found. At the genus level, *Bacteroides*, *Akkermansia*, *Octadecabacter*, *Parabacteroides,* and *Staphylococcus* (*p* < 0.001 and *q* < 0.05) were also changed with feeding type. However, the relative abundance of *Bifidobacterium* was not different between groups (*p* = 0.617) (Figure 3C) as observed in the RT-PCR analysis.

**Figure 4:**
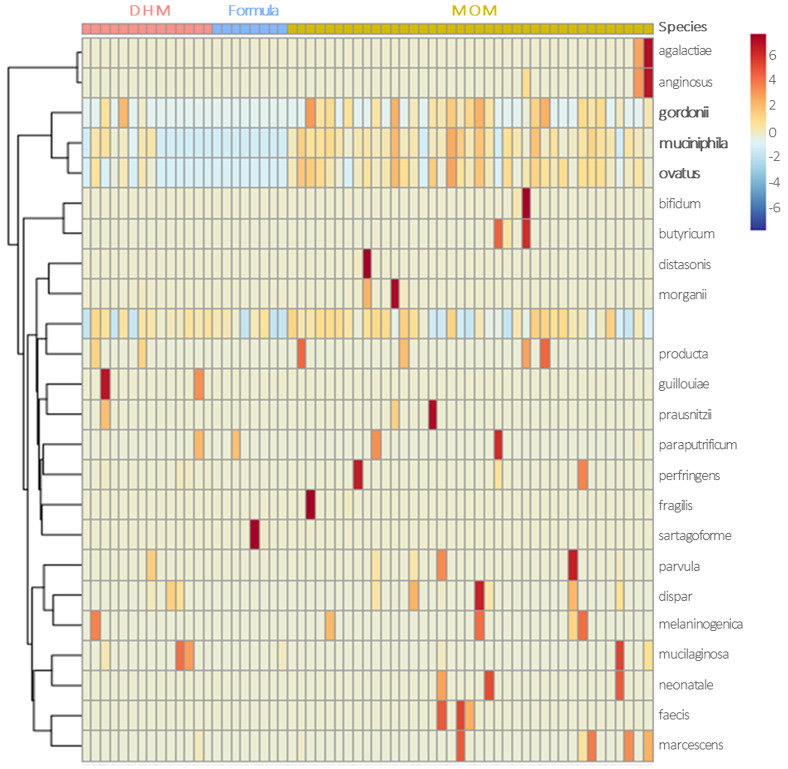
Distribution of bacterial species by feeding type: heatmap analysis shows clustering of bacterial species according to preterms’ feeding type (MOM, mother’s own milk; DHM, donor human milk; formula).

A heatmap analysis was performed between the relative abundance of bacterial species in fecal samples and infant types of feeding. In Figure 4, it is possible to observe that, contrary to formula, MOM and DHM were positively correlated with *Streptococcus gordonii*, *Akkermansia muciniphila,* and *Bacteroides ovatus* abundance (Figure 4).

### 3.4. Influence of Infant Feeding on Fecal Alkaline Phosphatase Activity

**Figure 5:**
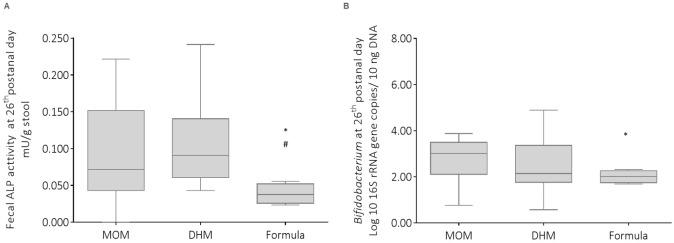
Influence of infant feeding received in the first week of life (MOM, mother’s own milk; DHM, donor human milk; formula) on (**A**) alkaline phosphatase (ALP) activity and (**B**) *Bifidobacterium* in fecal samples collected at the 26th postnatal day from preterm neonates. * MOM vs. formula; ^#^ DHM vs. formula. Differences were considered statistically significant when *p* < 0.05.

To explore how the type of infant feeding modulates ALP activity, ALP activity was measured in fecal samples collected from very preterm neonates at the 26th postnatal day. No differences were observed in the fecal ALP activity according to the predominant feeding type that neonates received during the 26 days of the study. However, we noticed that the type of infant feeding received in the sensitive window, that is the first week of life, impacted the activity of ALP in the fourth week of life (26th postnatal day). Very preterm neonates fed with MOM or DHM during the first week of life had significantly increased ALP activity at the 26th day of life, compared to that of formula fed neonates (*p* = 0.007 and *p* = 0.002, respectively) (Figure 5A). Curiously, MOM and DHM administration in the first week of life also tended to increase *Bifidobacterium* content at the 26th postnatal day (Figure 5B).

### 3.5. Impact of MOM in Different Proportions

The differences between MOM and formula groups were clear. However, the differences between MOM and DHM fed infants’ microbiota and ALP activity were not so clear. To determine the effect of different MOM proportions on the microbiota profile, we divided the infants fed predominantly with MOM and DHM into six groups: 100% to ≥90% (*n* = 20); <90% to ≥80% (*n* = 14); <80% to ≥70% (*n* = 18); <70% to ≥50% (*n* = 18); <50% to ≥35% (*n* = 10); and <35% (*n* = 15). Taking as the reference group the one that received 100% to ≥90% of MOM, microbial richness (Chao1 index) was lower in infants fed with <50% to ≥35% of MOM, when adjusted for a multivariable-adjusted model (Table 4). Significant differences were found in the group with a lower percentage of MOM (<35%) in the total amount of bacteria and *Firmicutes* when adjusted for the same model (Table 4). Neonates fed with less than 50% of MOM had lower amounts of *Bifidobacterium* (Table 4).

No differences were found in the ALP activity at the 26th postnatal day regarding the mentioned groups with different proportions of MOM (data not shown).

## 4. Discussion

In this prospective study, our results are in accordance with previous studies showing that MOM and DHM have a better impact on the intestinal microbiota of preterm infants [28] when compared to formula. In addition, we showed for the first time that the type of feeding received in the first week of life influences ALP activity. Finding that feeding type can modulate ALP may be of major importance as this enzyme reduces metabolic endotoxemia [11] and may protect against NEC. However, our study did not include neonates with NEC to explore this issue.

In our cohort, very preterm neonates were fed with MOM, DHM, or formula. Neonates fed predominantly with formula showed increased weight gain during the first 26 days of life. At first glance, it seems that formula promoted a greater growth of preterm neonates when compared to that of MOM or DHM. However, MOM and DHM were preferably administrated to premature neonates with lower weight, length, and cephalic perimeter at birth and, therefore, were more immature with compromised development, explaining why these neonates gained less weight during the study period and had more days of antibiotherapy. In addition, we found that formula promoted lower levels of *Bifidobacterium* and *Bacteroides* when compared to that of MOM (Figure 1). These findings suggest that formula delays colonization by anaerobic bacteria and, most likely, intestinal maturation. This is an expected result since *Bifidobacterium*, *Bacteroides,* and other anaerobes species use human milk oligosaccharides (HMOs) as a substrate [29] and these were absent in the formulas used at the time in our NICU. Even when adjusted for gestational age, mode of delivery, somatometry at birth, and neonatal antibiotherapy (factors known to strongly affect the establishment of initial microbiota [30]), we found that neonates fed predominantly with MOM had more absolute abundance of total bacteria, *Firmicutes,* and *Bifidobacterium* when compared to those mainly fed with formula. Microbial richness and *Firmicutes* content were significantly lower when the feedings contained less than 50% of MOM, when controlled for the same factors. However, no differences were observed in *Bifidobacterium* in the groups that received different proportions of MOM. The effects of DHM in very preterm neonates’ microbiota profile were less accentuated than those of formula, which could be explained by the similar composition in HMOs between MOM and DHM [31].

Regarding the sequencing data, we noticed that the results differed from those obtained by RT-PCR. Although this is a more complete approach, the analysis is based on relative abundances, instead of absolute bacterial quantification, which may explain the discrepancies found with respect to the species of *Bifidobacterium*. However, it allowed us to see other finding: human milk (MOM and DHM) promoted higher amounts of *Bacteroides ovatus* and *Akkermancia muciniphila* than did formula. Both these bacteria are beneficial for the intestines of preterm neonates. *B. ovatus* was described to be capable of inducing high mucosal IgA production when gnotobiotic mice were colonized with this strain compared to that when they were colonized with others strains [32]. Reduced levels of *A. muciniphila* have been associated with inflammatory bowel disease and metabolic disorders [33]. In neonates, reduced levels of *A. muciniphila* was found in extensive intestinal ischemia [34]. Actually, this mucin-degrading bacteria has been suggested to be a probiotic with promising effects on inflammation prevention [35]. Due to structural similarities of mucin with HMOs, *A. muciniphila* is able to digest HMOs [36].

MOM promoted increased bacterial richness compared to that of DHM and formula. In fact, it is possible to see a tendency towards a ladder effect favorable to MOM, as if it were a dose-response regarding the preobiotic and probiotic content (Figure 2A). However, no statistically significant differences were found in microbiota profiles between the groups (Figure 3A). This lack of difference can be explained by the discrepant number of individuals in each group (MOM = 39, DHM = 14, Formula = 8).

It is important to note that the clinical characteristics of the neonates included in MOM vs formula groups were significant different (gestational age, mode of delivery and somatometry at birth). However, despite these limitations, our results highlighted the importance of MOM administration in the first weeks of life on the intestinal microbiota development.

Besides having a beneficial microbial composition, MOM-fed neonates also showed increased fecal ALP activity. To the best of our knowledge, the present study analyzed for the first time the influence of neonate feeding in ALP activity in human very preterm neonates. Since the first week of life is considered to be a very important window to modulate gut microbiota of neonates, we explored how the type of feeding during this time-specific period influenced the ALP activity in fecal samples collected from very preterm neonates. We found that neonates fed with MOM and DHM during the first week of life had increased levels of ALP activity in fecal samples at postnatal day 26, compared to that of formula-fed neonates in the same period. Although human milk is an exogenous source of ALP [13], pasteurization destroys 99% of ALP present in the human milk [37]. Thus, we propose that the effect of human milk on the neonatal activity of ALP is not only due an intake of exogenous ALP, but also to an induction of the enzyme by some agent in both non-pasteurized and pasteurized human milk, and the agent is absent in neonatal formula. In fact, no differences were found in ALP activity between preterm rat pups that received formula with or without ALP supplementation [38,39,40]. Several molecules have been described to be involved in the induction of ALP activity such as omega-3, whey protein, or the short chain fatty-acid butyrate [41,42,43]. As discussed earlier, the presence of HMOs promote the growth of *Bifidobacterium* genus that have a role in neonatal intestinal maturation and immune tolerance [44] and are described as lipopolysaccharide (LPS)-suppressing bacteria [41]. During carbohydrate fermentation of HMOs present in human milk, *Bifidobacterium* enhances butyrate-producing bacteria by producing acetate that is used as a co-substrate for butyrate synthesis [45]. In addition, butyrate has been shown to increase ALP activity in an intestinal in vitro model [42]. Therefore, this could be a mechanism behind the *Bifodobacterium* correlation with ALP activity.

## 5. Conclusions

Overall, these findings point out the importance of MOM and DHM since both increase the levels beneficial bacteria, such as *Bifidobacterium*, *Bacteroides ovatus,* and *Akkermancia municiphila*. Moreover, our results showed that human milk intake during the first week of life increased the levels of *Bifidobacterium* and ALP activity measured at the fourth week of life (26th postnatal day). Since ALP activity, by dephosphorylating LPS, may prevent the initiation of signaling cascades that trigger inflammation, avoiding LPS binding to toll-like receptor, we believe that the first week of life is a sensitive window of opportunity to prevent metabolic endotoxemia and to protect against NEC. Future studies are needed to better understand the potential mechanism raised in this study by which oligosaccharides present in MOM and DHM may protect against NEC through ALP activity.

## Figures and Tables

**Table 1:** Primer sequences used for gut microbiota analysis.

Target Group	Primer Sequence (5′-3′)	Genomic DNA Standard	AT	Ref.
Total bacteria	AAA CTC AAA KGA ATT GAC GG CTC ACR RCA CGA GCT GAC	*Bacteroides vulgatus* ATCC 8482	62 °C	[22]
*Bacteroidetes*	CAT GTG GTT TAA TTC GAT GAT AGC TGA CGA CAA CCA TGC AG	*Bacteroides vulgatus* ATCC 8482	60 °C	[16]
*Firmicutes*	ATG TGG TTT AAT TCG AAG CA AGC TGA CGA CAA CCA TGC AC	*Lactobacillus gasseri* ATCC 33323	60 °C	[16]
*γ-Proteobacteria*	TCGTCAGCTCGTGTYGTGA CGTAAGGGCCATGATG	*E. coli* ATCC 25922	61 °C	[22]
*Lactobacillus*	GAG GCA GCA GTA GGG AAT CTT C GGC CAG TTA CTA CCT CTA TCC TTC TTC	*Lactobacillus gasseri* ATCC 33323	60 °C	[16]
*Bifidobacterium*	CGC GTC YGG TGT GAA AG CCC CAC ATC CAG CAT CCA	*Bifidobacterium longum* ATCC 15697	60 °C	[16]
*Bacteroides*	ATA GCC TTT CGA AAG RAA GAT CCA GTA TCA ACT GCA ATT TTA	*Bacteroides vulgatus* ATCC 33563	60 °C	[16]
*Enterococcus*	CCC TTA TTG TTA GTT GCC ATC ATT ACT CGT TGT ACT TCC CT TGT	*Enterococcus gilvus* ATCC BAA-350	61 °C	[16]
*Escherichia coli*	GTA AGT TAC ACT ATA AAA GCA CCG TCG TCT GTG TGG ATG GTA ATA AAT TTT TG	*Escherichia coli* ATCC 25922	60 °C	[23]

AT, annealing temperature.

**Table 2:** Clinical data of very preterm neonates receiving different types of infant feeding during study period.

	MOM (*n* = 75)	DHM (*n* = 20)	Formula (*n* = 13)	*p*-Value (MOM vs. DHM)	*p*-Value (MOM vs. Formula)	*p*-Value (DHM vs. Formula)
Extremely/very preterm n	28/47	5/15	0/13	0.205	0.004	0.065
Gestational age (mean ± SD)	28.3 ± 2.1	28.6 ± 1.8	29.6 ±1.1	0.103	0.003	0.17
Vaginal delivery/C-section, n	34/41	7/13	1/12	0.407	0.010	0.074
Somatometry at birth (mean ± SD)						
Weight, g	1123 ± 345	1173 ± 284	1416 ± 219	0.438	0.002	0.011
Length, cm	36.2 ± 3.3	36.8 ± 3.2	39.1 ± 2.0	0.428	0.003	0.027
Cephalic perimeter, cm	25.4 ± 2.4	25.3 ± 1.8	27.2 ± 1.5	0.996	0.007	0.008
Z-score						
Weight at birth	−0.27 ± 0.82	0.04 ± 1.45	0.15 ± 0.70	0.977	0.057	0.789
Length at birth	−0.44 ± 1.09	−0.25 ± 1.61	0.04 ±0.65	0.777	0.051	0.543
Cephalic perimeter at birth	−0.46 ± 0.90	−0.68 ± 1.79	−0.77 ± 1.28	0.731	0.147	0.331
Weight at 26th	−0.08 ± 1.85	0.85 ± 1.62	1.65 ± 1.13	0.257	0.001	0.132
Δ weight until 26th day, g	200 ± 190	200 ± 170	420 ± 160	0.671	<0.001	0.001
Postnatal day of full enteral nutrition (mean ± SD)	16.5 ± 8.5	16.4 ± 5.6	9.5 ± 4.2	0.401	0.002	<0.001
Days of antibiotherapy (mean ± SD)	12 ± 11	13 ± 8	5 ± 3	0.178	0.010	<0.001
Days of hospitalization (mean ± SD)	58 ± 24	59 ± 21	42 ± 12	0.874	0.026	0.018

MOM, mother’s own milk. DHM, donor human milk.

**Table 3:** Association between neonates feeding (independent variable) and neonates’ microbiota at the 26th postnatal day (dependent variables).

	MOM	DHM		Formula	
		95% CI	*p*-Value	95% CI	*p*-Value
Total Bacteria					
Gestational age-adjusted	1 (referent)	−0.556 (−1.093 to −0.079)	0.023	−0.889 (−1.341 to −0.438)	<0.001
Mode of delivery-adjusted	1 (referent)	−0.565 (−1.036 to −0.095)	0.018	−0.552 (−1.047 to −0.057)	0.029
Multivariable-adjusted ^a^	1 (referent)	−0.659 (–1.082 to −0.236)	0.002	−0.721 (−1.158 to −0.284)	0.001
*Firmicutes*					
Gestational age-adjusted	1 (referent)	−0.585 (−1.183 to 0.013)	0.055	−0.789 (−1.322 to −0.256)	0.004
Mode of delivery-adjusted	1 (referent)	−0.587 (−1.161 to −0.013)	0.045	−0.456 (−1.060 to 0.162)	0.139
Multivariable-adjusted ^a^	1 (referent)	−0.610 (–1.114 to −0.107)	0.017	−0.759 (−1.279 to −0.238)	0.004
*Bifidobacterium*					
Gestational age-adjusted	1 (referent)	−2.002 (−3.315 to −0.689)	0.003	−2.092 (−3.262 to −0.922)	<0.001
Mode of delivery-adjusted	1 (referent)	−0.291 (−1.016 to 0.434)	0.431	−0.684 (−1.447 to 0.079)	0.079
Multivariable-adjusted ^a^	1 (referent)	−0.656 (−1.388 to 0.075)	0.079	−0.975 (−1.716 to −0.233)	0.010

^a^ Multivariable-adjusted model included gestational age, mode of delivery, Z-scores’ growth parameters (weight, length, and cephalic perimeter at birth), and infant’s antibiotherapy received within 8 days prior to fecal collection. MOM, mother’s own milk. DHM, donor human milk.

**Table 4:** Association between different proportions of MOM (independent variable) and neonates’ microbiota at the 26th postnatal day (dependent variables).

	*	<90% to ≥80%		<80% to ≥70%		<70% to ≥50%		<50% to ≥35%		<35%	
		95% CI	*p*	95% CI	*p*	95% CI	*p*	95% CI	*p*	95% CI	*p*
Chao1 index											
Multivariable -adjusted ^a^	Ref.	2.559 (–0.707 to 5.824)	0.125	−2.384 (–5.749 to 0.980)	0.165	1.603 (–2.467 to 5.673)	0.440	−9.451 (−15.660 to−3.242)	0.003	−2.820 (–6.273 to 0.634)	0.110
Total Bacteria											
Multivariable -adjusted ^a^	Ref.	−0.173 (–0.697 to 0.351)	0.518	−0.179 (–0.684 to 0.326)	0.487	0.104 (–0.355 to 0.562)	0.658	−0.611 (–1.235 to 0.12)	0.055	−0.771 (–1.311 to −0.231)	0.005
*Firmicutes*											
Multivariable -adjusted ^a^	Ref.	−0.551 (–1.165 to 0.063)	0.079	−0.502 (–1.094 to 0.090)	0.097	−0.401 (–0.939 to 0.137)	0.144	−1.297 (–2.028 to −0.566)	0.001	−1.115 (–1.749 to −0.482)	0.001
*Bifidobacterium*											
Multivariable -adjusted ^a^	Ref.	0.605 (−0.495 to 1.704)	0.281	−0.039 (−1.018 to 1.096)	0.943	−0.237 (−1.196 to 0.723)	0.628	0.207 (−1.078 to 1.492)	0.753	−0.424 (−1.543 to 0.696)	0.458

* Reference category: 100% to ≥90% of MOM ^a^ Multivariable-adjusted model included gestational age, mode of delivery, Z-scores’ growth parameters (weight, length, and cephalic perimeter at birth), and infant’s antibiotherapy received within 8 days prior to fecal collection. MOM, mother’s own milk.

## Data Availability

The data presented in this study are available on request from the corresponding author.

## References

[1] Hill C.J., Lynch D.B., Murphy K., Ulaszewska M., Jeffery I.B., O’Shea C.A., Watkins C., Dempsey E., Mattivi F., Tuohy K. (2017). Evolution of gut microbiota composition from birth to 24 weeks in the INFANTMET Cohort. Microbiome.

[2] Van Belkum M., Alvarez L.M., Neu J. (2019). Preterm neonatal immunology at the intestinal interface. Cell. Mol. Life Sci..

[3] Xu W., Judge M.P., Maas K., Hussain N., McGrath J.M., Henderson W.A., Cong X. (2018). Systematic Review of the Effect of Enteral Feeding on Gut Microbiota in Preterm Infants. J. Obstet. Gynecol. Neonatal Nurs..

[4] Kumar R.K., Singhal A., Vaidya U., Banerjee S., Anwar F., Rao S. (2017). Optimizing Nutrition in Preterm Low Birth Weight Infants—Consensus Summary. Front. Nutr..

[5] Agostoni C., Braegger C., Decsi T., Kolacek S., Koletzko B., Michaelsen K.F., Mihatsch W., A Moreno L., Puntis J., Shamir R. (2009). Breast-feeding: A Commentary by the ESPGHAN Committee on Nutrition. J. Pediatr. Gastroenterol. Nutr..

[6] Korpela K., Blakstad E.W., Moltu S.J., Strømmen K., Nakstad B., Rønnestad A.E., Brække K., Iversen P.O., Drevon C.A., De Vos W. (2018). Intestinal microbiota development and gestational age in preterm neonates. Sci. Rep..

[7] Arslanoglu S., Willemijn C., Guido M., Christian B., Cristina C., Virginie C., Tamas D., Magnus D., Mary F., Iva H. (2013). Donor human milk for preterm infants: Current evidence and research directions. J. Pediatr. Gastroenterol. Nutr..

[8] Quigley M., Henderson G., My A., Mcguire W. (2007). Formula milk versus donor breast milk for feeding preterm or low birth weight infants (Review). Cochrane Database Syst. Rev..

[9] Cheong J.L.Y., Burnett A.C., Treyvaud K., Spittle A.J. (2019). Early environment and long-term outcomes of preterm infants. J. Neural Transm..

[10] Neu J. (2020). Necrotizing Enterocolitis: A Multi-omic Approach and the Role of the Microbiome. Dig. Dis. Sci..

[11] Rader B.A. (2017). Alkaline Phosphatase, an Unconventional Immune Protein. Front. Immunol..

[12] Heath M., Buckley R., Gerber Z., Davis P., Linneman L., Gong Q., Barkemeyer B., Fang Z., Good M., Penn D. (2019). Association of Intestinal Alkaline Phosphatase With Necrotizing Enterocolitis Among Premature Infants. JAMA Netw. Open.

[13] Yang Y., Rader E., Peters-Carr M., Bent R.C., Smilowitz J.T., Guillemin K., Rader B. (2019). Ontogeny of alkaline phosphatase activity in infant intestines and breast milk. BMC Pediatr..

[14] Morais J., Marques C., Teixeira D., Durão C., Faria A., Brito S., Cardoso M., Macedo I., Tomé T., Calhau C. (2019). FEEDMI: A Study Protocol to Determine the Influence of Infant-Feeding on Very-Preterm-Infant’s Gut Microbiota. Neonatology.

[15] Fenton T.R., Kim J.H. (2013). A systematic review and meta-analysis to revise the Fenton growth chart for preterm infants. BMC Pediatr..

[16] Marques C., Meireles M., Norberto S., Leite J., Freitas J., Pestana D., Faria A., Calhau C. (2015). High-fat diet-induced obesity Rat model: A comparison between Wistar and Sprague-Dawley Rat. Adipocyte.

[17] Gale C., Logan K.M., Santhakumaran S., Parkinson J.R.C., Hyde M.J., Modi N. (2012). Effect of Breastfeeding compared with Formula Feeding on Infant Body Composition: A Systematic Review and Meta-Analysis. Am. J. Clin. Nutr..

[18] Guaraldi F., Salvatori G. (2012). Effect of Breast and Formula Feeding on Gut Microbiota Shaping in Newborns. Front. Cell. Infect. Microbiol..

[19] Mshvildadze M., Neu J., Shuster J., Theriaque D., Li N., Mai V. (2010). Intestinal Microbial Ecology in Premature Infants Assessed with Non–Culture-Based Techniques. J. Pediatr..

[20] Gregory K.E., Samuel B.S., Houghteling P., Shan G., Ausubel F.M., Sadreyev R.I., Walker W.A. (2016). Influence of maternal breast milk ingestion on acquisition of the intestinal microbiome in preterm infants. Microbiome.

[21] Morais J., Marques C., Teixeira D., Durão C., Faria A., Brito S., Cardoso M., Macedo I., Pereira E., Tomé T. (2020). Extremely preterm neonates have more Lactobacillus in meconium than very preterm neonates – the in utero microbial colonization hypothesis. Gut Microbes.

[22] De Gregoris T.B., Aldred N., Clare A.S., Burgess J.G. (2011). Improvement of phylum- and class-specific primers for real-time PCR quantification of bacterial taxa. J. Microbiol. Methods.

[23] Waitzberg D.L., Pereira C.C.A., Logullo L., Jacintho T.M., Almeida D., Da Silva M.L.T., Torrinhas R.S.M.D.M. (2012). Microbiota benefits after inulin and partially hydrolized guar gum supplementation: A randomized clinical trial in constipated women. Nutr. Hosp..

[24] Walters W., Hyde E.R., Berg-Lyons D., Ackermann G., Humphrey G., Parada A., Gilbert J.A., Jansson J.K., Caporaso J.G., Fuhrman J.A. (2015). Improved Bacterial 16S rRNA Gene (V4 and V4-5) and Fungal Internal Transcribed Spacer Marker Gene Primers for Microbial Community Surveys. mSystems.

[25] Calhau C., Martel F., Hipólito-Reis C., Azevedo I. (2000). Differences between duodenal and jejunal rat alkaline phosphatase. Clin. Biochem..

[26] Malo M.S. (2015). A High Level of Intestinal Alkaline Phosphatase Is Protective Against Type 2 Diabetes Mellitus Irrespective of Obesity. EBioMedicine.

[27] Chong J., Liu P., Zhou G., Xia J. (2020). Using MicrobiomeAnalyst for comprehensive statistical, functional, and meta-analysis of microbiome data. Nat. Protoc..

[28] Parra-Llorca A., Gormaz M., Alcántara C., Cernada M., Nuñez-Ramiro A., Vento M., Collado M.C. (2018). Preterm Gut Microbiome Depending on Feeding Type: Significance of Donor Human Milk. Front. Microbiol..

[29] German J.B., Freeman S.L., Lebrilla C.B., Mills D.A. (2008). Human milk oligosaccharides: Evolution, structures and bioselectiivty as substrates for intestinal bacteria. Pers. Nutr. Divers. Needs Infants Child..

[30] Arboleya S., Sánchez B., Milani C., Duranti S., Solís G., Fernández N., Reyes-Gavilán C.G.D.L., Ventura M., Margolles A., Gueimonde M. (2015). Intestinal Microbiota Development in Preterm Neonates and Effect of Perinatal Antibiotics. J. Pediatr..

[31] Marx C., Bridge R., Wolf A.K., Rich W., Kim J.H., Bode L. (2013). Human Milk Oligosaccharide Composition Differs between Donor Milk and Mother’s Own Milk in the NICU. J. Hum. Lact..

[32] Yang C., Mogno I., Contijoch E.J., Borgerding J.N., Aggarwala V., Li Z., Siu S., Grasset E.K., Helmus D.S., Dubinsky M.C. (2020). Fecal IgA Levels Are Determined by Strain-Level Differences in Bacteroides ovatus and Are Modifiable by Gut Microbiota Manipulation. Cell Host Microbe.

[33] Derrien M., Belzer C., de Vos W.M. (2017). Akkermansia muciniphila and its role in regulating host functions. Microb. Pathog..

[34] Romani L., Del Chierico F., Chiriaco M., Foligno S., Reddel S., Salvatori G., Cifaldi C., Faraci S., Finocchi A., Rossi P. (2020). Gut Mucosal and Fecal Microbiota Profiling Combined to Intestinal Immune System in Neonates Affected by Intestinal Ischemic Injuries. Front. Cell. Infect. Microbiol..

[35] Bavineni M., Wassenaar T.M., Agnihotri K., Ussery D.W., Lüscher T.F., Mehta J.L. (2019). Mechanisms linking preterm birth to onset of cardiovascular disease later in adulthood. Eur. Heart J..

[36] Ottman N. (2015). Host Immunostimulation and Substrate Utilization of the Gut Symbiont Akkermansia Muciniphila. Ph.D. Thesis.

[37] Neu J. (2019). Mother’s Own Milk: How Does It Differ from Donor Milk for the Baby. Breastfeed. Med..

[38] Heinzerling N.P., Liedel J.L., Welak S.R., Fredrich K., Biesterveld B.E., Pritchard K.A., Gourlay D.M. (2014). Intestinal alkaline phosphatase is protective to the preterm rat pup intestine. J. Pediatr. Surg..

[39] Biesterveld B.E., Koehler S.M., Heinzerling N.P., Rentea R.M., Fredrich K., Welak S.R., Gourlay D.M. (2015). Intestinal alkaline phosphatase to treat necrotizing enterocolitis. J. Surg. Res..

[40] Rentea R., Rentea M., Biesterveld B., Liedel J., Gourlay D. (2018). Factors Known to Influence the Development of Necrotizing Enterocolitis to Modify Expression and Activity of Intestinal Alkaline Phosphatase in a Newborn Neonatal Rat Model. Eur. J. Pediatr. Surg..

[41] Kaliannan K., Wang B., Li X.-Y., Kim K.-J., Kang J.X. (2015). A host-microbiome interaction mediates the opposing effects of omega-6 and omega-3 fatty acids on metabolic endotoxemia. Sci. Rep..

[42] Gonçalves P., Catarino T.A., Gregório I., Martel F. (2012). Inhibition of butyrate uptake by the primary bile salt chenodeoxycholic acid in intestinal epithelial cells. J. Cell. Biochem..

[43] Navis M., Muncan V., Sangild P.T., Willumsen L.M., Koelink P.J., Wildenberg M.E., Abrahamse E., Thymann T., Van Elburg R.M., Renes I.B. (2020). Beneficial Effect of Mildly Pasteurized Whey Protein on Intestinal Integrity and Innate Defense in Preterm and Near-Term Piglets. Nutrients.

[44] Underwood M.A., Gaerlan S.C., De Leoz M.L.A., Dimapasoc L.M., Kalanetra K.M., Lemay D.G., German J.B., Mills D.A., Lebrilla C.B. (2015). Human milk oligosaccharides in premature infants: Absorption, excretion, and influence on the intestinal microbiota. Pediatr. Res..

[45] Rivière A., Selak M., Lantin D., Leroy F., De Vuyst L. (2016). Bifidobacteria and Butyrate-Producing Colon Bacteria: Importance and Strategies for Their Stimulation in the Human Gut. Front. Microbiol..

